# Association of phenotypic and functional characteristics of regulatory T cells from minor salivary glands with expression of the delta 7 FoxP3 splice variant in patients with Sjögren’s disease

**DOI:** 10.3389/fimmu.2026.1959200

**Published:** 2026-10-02

**Authors:** Varvara G. Blinova, Yulia A. Gladilina, Ekaterina B. Rodionova, Vladimir I. Vasilyev, Natalia S. Shornikova, Anastasiya N. Shishparenok, Dmitry D. Zhdanov

**Affiliations:** 1 Laboratory of Medical Biotechnology, Institute of Biomedical Chemistry, Moscow, Russia; 2 DDM Clinic, Moscow, Russia; 3 Dr. Laskov’s Clinic, Moscow, Russia; 4 Department of Biochemistry, Peoples’ Friendship University of Russia named after Patrice Lumumba (RUDN University), Moscow, Russia

**Keywords:** alternative splicing, FoxP3, proliferation, regulatory T cells, Sjögren’s disease, splice variants, suppressive activity

## Abstract

**Background:**

Sjögren’s disease (SjD) is a systemic autoimmune disorder characterized by lymphoplasmacytic infiltration of exocrine glands, including minor salivary glands (MSG), with potential formation of ectopic germinal centers. Regulatory T cells (Tregs) are critical suppressors of autoimmune responses, and their dysfunction has been implicated in SjD pathogenesis. FoxP3, the master transcription factor of Tregs, undergoes alternative splicing to produce four variants: full-length (FL), Δ2, Δ7, and Δ2Δ7. While FoxP3 splice variants are known to influence Treg proliferation and suppressive activity, their profile and functional significance in Tregs from SjD patients remain poorly characterized.

**Methods:**

Peripheral blood was collected from 20 SjD patients or 20 healthy donors (HD). MSG biopsies were obtained from 10 SjD patients. Tregs were isolated from blood and biopsy tissue by immunomagnetic separation and expanded ex vivo. FoxP3 splice variant mRNA levels were quantified by real-time RT-PCR. Treg phenotype (CD4, CD25, CD127, CD152, Helios), suppressive molecules (CD39, CD223, granzymes A/B, perforin), was assessed by flow cytometry. The concentrations of suppressive cytokines IL-10 andIL-35 were accessed by Bio-Plex assay. Proliferation was evaluated by cell counting. Suppressive activity was determined by mixed lymphocyte reaction. Splice-switching oligonucleotides (SSOs) were used to induce Δ7 FoxP3 expression to confirm direct functional effects.

**Results:**

Peripheral blood Tregs from SjD patients showed significantly decreased increased Δ7 FoxP3 splice variant compared to HD. MSG-derived Tregs from SjD patients also predominantly expressed the Δ7 FoxP3 variant. High-expressing Δ7 FoxP3 Tregs exhibited reduced proliferative activity, division frequency and decreased suppressive activity compared to low-expressing Δ7 FoxP3 Tregs. High-expressing Δ7 FoxP3 Tregs showed significantly reduced CD127low and Helios expression, decreased CD39, CD223, granzyme, and granzyme B. IL-10 and IL-35 production was also reduced. SSO-mediated induction of Δ7 FoxP3 MSG-derived Tregs confirmed these phenotypic and functional changes.

**Conclusions:**

This study demonstrates that elevated Δ7 FoxP3 splice variant expression in MSG-derived Tregs from SjD patients is associated with impaired proliferative and suppressive function, reduced expression of suppressive molecules and cytokines, and a phenotype suggestive of a transitional or immature Treg state. The results highlight the functional consequences of altered FoxP3 alternative splicing in SjD pathogenesis and suggest a possible involvement of Tregs in the biology of ectopic germinal centers in SjD.

## 1 Introduction

Alternative splicing of Forkhead box protein P3 (FoxP3) pre-mRNA, which results in the appearance of four splice variants—the full-length (FL) variant, the variant with deleted exon 2 (Δ2), the variant with deleted exon 7 (Δ7), and the variant with two deleted exons 2 and 7 (Δ2Δ7) has been observed in various pathological conditions (1–3). Since FoxP3 is the main transcriptional factor that regulates the homeostasis of regulatory T cells (Tregs), its alternative splicing has become a major focus in the context of autoimmune diseases (4–6). Previously, abnormal changes in FoxP3 splice variant expression have been identified in peripheral Tregs from patients with multiple sclerosis and amyotrophic lateral sclerosis (7, 8).

Sjögren’s disease (SjD) is a systemic autoimmune and lymphoproliferative disorder characterized by lymphoplasmacytic infiltration of various tissues and organs as a result of loss of self-tolerance (9). With exocrine glands (such as salivary, lacrimal, and sweat glands) being primary targets, infiltration may also cause extraglandular manifestations affecting the joints, blood vessels, lungs, kidneys, reticuloendothelial system, and gastrointestinal and nervous systems (10). Identification of lymphoplasmacytic infiltration in minor salivary gland (MSG) biopsy is one of the key diagnostic markers of the disease (11). One of the hallmarks of SjD is the development of mucosa-associated lymphoid tissue lymphomas and diffuse large B-cell lymphomas (12). These may arise in the course of the disease due to inadequate activation and proliferation of T and B lymphocytes, leading to the formation of ectopic lymphoid structures. Considering that Tregs are the main immune cells that suppress the activity of activated lymphocytes, a decrease in their number and/or insufficiency of their functional activity, which has been described previously (13), may contribute to the pathogenesis of SjD. The reasons underlying impaired Treg functionality in SjD are being extensively studied.

Despite the growing number of publications on the association of FoxP3 splice variants with physiological and pathological conditions, their roles in the onset and progression of autoimmune diseases, and particularly SjD, remain not fully understood. In this work, we aimed to determine the expression levels of FoxP3 splice variants in Tregs isolated from peripheral blood and MSG biopsies of SjD patients. We also aimed to study the proliferation, immunophenotype, and suppressive activity of MSG Tregs with high and low expression of the Δ7 FoxP3 splice variant.

## 2 Materials and methods

### 2.1 Study participants, biopsy and blood sampling

Study subjects were recruited at the Dr. Laskov’s Clinic and DDM Clinic (Moscow, Russian Federation). All patients and healthy donors (HD) provided written informed consent to participate in the study. The study (No. 1/1 of April 18, 2025) was approved by the ethical group of the Sjögren’s Disease Scientific and Educational Center and was conducted in accordance with the Declaration of Helsinki of 1964 and its subsequent updates. Only adult participants (aged 18 and older) were enrolled in the study. Patients with hepatitis C (HCV) were excluded, as extrahepatic manifestations of HCV may resemble the clinical and immunological features of SjD. Prior to biopsy and blood sampling, each patient underwent a detailed physical examination. The diagnosis was established based on information obtained during patient interview, medical history (anamnesis), physical examination findings, and results of instrumental and laboratory studies. Peripheral blood samples were collected from a cohort of 20 patients diagnosed with SjD. A biopsy was performed in a group of 10 patients. The diagnosis of SjD was established using the European Alliance of Associations for Rheumatology (EULAR)/American College of Rheumatology (ACR) criteria (2016) (14). Disease activity was evaluated according to the EULAR Sjögren’s Syndrome Disease Activity Index (ESSDAI) (15). Based on ESSDAI scores, patients were classified as having low, moderate, or high disease activity.

The control group consisted of 20 age- and sex-matched healthy subjects. Physical examination and laboratory studies were performed for all subjects enrolled in the healthy control group to exclude any underlying rheumatological conditions. **Table 1** summarizes the main demographic characteristics of the study participants who underwent peripheral blood sampling. Peripheral blood from SjD patients and HD was collected into vacuum tubes containing the anticoagulant K3EDTA via venipuncture.

**Table 1:** Demographics of SjD patients and healthy donors.

Parameter	SjD group (n = 20)	HD group (n = 20)
Number of female patients (%)	17 (85%)	12 (62.8%)
Age at study enrollment, mean ± SD*	45.3 ± 14.3	36.2 ± 12.2
Age range	21 – 72	21 – 58

*SD, standard deviation (95% confidence interval).

The main clinical characteristics of the SjD group are provided in **Table 2**. All data are presented as the mean ± SD, 95% confidence interval.

**Table 2:** Clinical data of SjD patients who underwent peripheral blood sampling.

Parameter	SjD group (n = 20)
Disease duration (years)	6.1 ± 3.8 (2 – 15)
ESSDAI, patients (%)	
• low	3 (15%)
• moderate	15 (75%)
• high	2 (10%)
Extraglandular manifestations, patients (%)	
• skin	2 (10%)
• hematological	4 (20%)
• lymph nodes	1 (5%)
• blood vessels	5 (25%)
• hepatobiliary system	1 (5%)
• musculoskeletal system	8 (40%)
• reproductive system	1 (5%)

All data are presented as the mean ± SD, 95% confidence interval.

Disease duration was defined as the time from the onset of first symptoms to study enrollment. The mean disease duration was 6.1 ± 3.8 years. Among all patients in the SjD group, disease activity assessed by the ESSDAI was low in 3 (15%) cases, moderate in 15 (75%) cases, and high in 2 (10%) cases. Two subjects with high disease activity developed lymphoproliferative complications: one patient had generalized MALT lymphoma of the parotid, submandibular, and sublingual salivary glands, and one had MALT lymphoma of the parotid gland. Fifteen patients (75%) exhibited extraglandular manifestations of SjD. Systemic involvement affected the skin (severe dryness, exanthema), the vascular system (Raynaud’s syndrome, hemorrhagic vasculitis), the hepatobiliary system (autoimmune cholangitis), and the musculoskeletal system (osteoarthritis, arthritis, arthralgia, Heberden’s nodes). Hematological manifestations included thrombocytopenia and neutropenia. Lymph node enlargement was observed in one case. One patient had a history of three miscarriages. Among all SjD patients, 14 subjects had never received any therapy for SjD. Two years prior to study enrollment, two patients had received prednisolone monotherapy, one had received hydroxychloroquine, and two had received a combination of hydroxychloroquine and prednisolone. Only one patient had received pathogenetic therapy with rituximab, with the last infusion administered two years prior to enrollment. Given that the included SjD patients presented with diverse clinical phenotypes and a highly variable disease duration (ranging from 2 to 15 years), this population likely represents significant immunopathological heterogeneity.

MSG biopsy was performed in 10 patients. A 0.5 cm longitudinal incision was made with a surgical scalpel in the mucous membrane of the lower lip, near the oral commissure. Prior to the incision, infiltration anesthesia was administered using 1 mL of 2% lidocaine hydrochloride. A conglomerate of MSGs, approximately 0.5 × 0.5 cm in size, was excised. Hemostasis was achieved with aminocaproic acid, and the wound was closed with catgut sutures.

The group of SjD patients who underwent biopsy was also heterogeneous. Age ranged from 21 to 72 years, with a mean of 46.3 ± 15.7 years. Among these participants, 7 subjects (70%) were female. The main clinical characteristics of this group are provided below in **Table 3**.

**Table 3:** Clinical data of SjD patients who underwent MSG biopsy.

Parameter	SjD group (n = 10)
Disease duration (years)	6.6 ± 5.2 (2 – 15)
ESSDAI, patients (%)	
• low	4 (40%)
• moderate	5 (50%)
• high	1 (10%)
Extraglandular manifestations, patients (%)	
• skin	1 (10%)
• hematological	4 (40%)
• blood vessels	2 (20%)
• hepatobiliary system	1 (10%)
• musculoskeletal system	3 (30%)

All data are presented as the mean ± SD, 95% confidence interval.

Disease duration was 6.6 ± 5.2 years. According to the ESSDAI, disease activity was low in 4 (40%) cases, moderate in 5 (50%) cases, and high in 1 (10%) case. One patient with high SjD activity developed generalized MALT lymphoma of the parotid, submandibular, and sublingual salivary glands. In seven patients (70%) of this group, extraglandular manifestations were observed. Among them, one patient had autoimmune cholangitis, two had hemorrhagic vasculitis, and laboratory findings in four patients revealed hematological alterations such as neutropenia and leukopenia. One patient presented with erythematous skin rashes, and three subjects suffered from arthralgia. Among this group, 7 patients (70%) had never received any therapy for SjD. Two patients had received a combination of hydroxychloroquine and prednisolone, which was discontinued two years prior to study enrollment. One patient had received chlorambucil more than two years prior to the study.

Blood from patients was collected by venipuncture into Vacuette vacuum tubes containing K3EDTA as an anticoagulant (Greiner Bio-One, Kremsmunster, Austria).

### 2.2 Hematoxylin and eosin staining of MSG biopsies

Formalin-fixed, paraffin-embedded tissue sections of MSG tissue were cut at 4 – 5 µm. Sections were deparaffinated in xylene and rehydrated through a graded ethanol to distilled water. For routine histomorphological evaluation, sections were stained with 0.1% Mayer’s hematoxylin (Sigma-Aldrich, St. Louis, MO, USA) for 10 min, rinsed in tap water, and differentiated in 0.5% acid alcohol for a few seconds, followed by bluing in Scott’s tap water substitute (Sigma-Aldrich, St. Louis, MO, USA). Slides were then counterstained with 0.5% eosin (Sigma-Aldrich, St. Louis, MO, USA) for 2 min, dehydrated through graded ethanol, dehydrated in xylene, and coverslipped with a permanent mounting medium (Thermo Fisher Scientific, Waltham, MA, USA). Stained sections were examined under a light microscope (Leica Microsystems, Wetzlar, Germany).

### 2.3 Isolation of Tregs from peripheral blood or biopsy material and ex vivo expansion

Isolation of Tregs from peripheral blood was performed by immunomagnetic cell separation using the CD4+CD25+ Treg Isolation Kit (Miltenyi Biotec, Bergisch Gladbach, Germany) following blood gradient centrifugation on Ficoll (1.077 g/mL, Paneco, Moscow, Russia) according to a previously described protocol (7). Salivary biopsy tissue from the operating room was transported on ice in 2 mL vials containing DMEM (Paneco, Moscow, Russia) with 15 mM HEPES supplemented with 5% fetal bovine serum (FBS, Capricorn Scientific, Ebsdorfergrund, Germany). Biopsy material was washed three times in sterile DPBS and incubated in 100 µL of Collagenase/Hyaluronidase (Stemcell Technologies, Vancouver, British Columbia, Canada) diluted in EpiCult-C Medium Human (Stemcell Technologies, Vancouver, British Columbia, Canada) at 37 °C for 12 h according to the manufacturer’s protocol to disrupt the extracellular matrix holding the cells together. Incubation was performed in a 5% CO_2_ incubator. After that, cells were resuspended by pipetting and subjected to gradient centrifugation on Ficoll. As described above, Tregs were isolated by immunomagnetic cell separation using the CD4+CD25+ Treg Isolation Kit (Miltenyi Biotec, Bergisch Gladbach, Germany).

### 2.4 Study of FoxP3 and FoxP3 splice variants expression

The expression levels of total FoxP3 mRNA and its splice variants were determined by real-time RT-PCR using the primers shown in **Table 4**. RNA isolation, reverse transcription, and amplification were performed as described previously (16, 17). Three genes: beta-actin, glyceraldehyde-3-phosphate dehydrogenase (GAPDH), and 18S, were used as reference genes, and FoxP3 mRNA levels were normalized to their average expression.

**Table 4:** List of primers used for real-time RT–PCR.

Target	Sense 5’ – 3’	Antisense 5’ – 3’	Tann, °C
FoxP3 total	agagagcctgcctcagtaca	tgacgctgcttctgtgtagg	60
FL FoxP3	ctagtcatggtggcaccctc	gatgaagccttggtcagtgcc	59
Δ2 FoxP3	cagctgcagctctcaacggtg	gatgaagccttggtcagtgcc	61
Δ7 FoxP3	ctagtcatggtggcaccctc	gtcggatgatgcctgctgctc	60
Δ2Δ7 FoxP3	cagctgcagctctcaacggtg	gtcggatgatgcctgctgctc	62
Beta-actin	cctgggcatggagtcctgt	atctccttctgcatcctgtcg	60
18S	ggatccattggagggcaagt	acgagctttttaactgcagcaa	62
GAPDH	gaaggtgaaggtcggagtc	gaagatggtgatgggatttc	57

### 2.5 Flow cytometric analysis of cell markers and suppressive molecules

The expression of Treg-associated cell markers was measured by flow cytometry using a MACSQuant Analyzer 10 flow cytometer (Miltenyi Biotec, Bergisch Gladbach, Germany). The expression of the membrane molecules CD4, CD25, and CD127 was assessed after cell labeling with the Treg Surface Marker Analysis Cocktail (Miltenyi Biotec, Bergisch Gladbach, Germany) or anti-CD25–APC. All antibodies used in this study were from Miltenyi Biotec (Bergisch Gladbach, Germany). To detect membrane-associated suppressive molecules, Tregs were labeled with anti-CD39–FITC or anti-CD223–APC. For intracellular Helios detection, Tregs were fixed with Fixation Buffer, permeabilized with Intracellular Staining Permeabilization Wash Buffer (both from BioLegend, Inc., San Diego, CA, USA), and labeled with anti-Helios-PE (Miltenyi Biotec, Bergisch Gladbach, Germany). Detection of granzymes A and B and perforin was performed after cell stimulation and subsequent labeling, as described previously (8).

Concentrations of IL-10 and IL-35 in Treg culture media were determined by flow cytometry–based Bio-Plex assay using the Bio-Plex 200 System (Bio-Rad, Hercules, CA, USA). Prior to analysis, media were processed with the 12-Plex Bio-Plex Pro™ Human Treg Cytokine Panel (Bio-Rad, Hercules, CA, USA) according to the manufacturer’s protocol.

### 2.6 Evaluation of proliferation intensity and suppressive activity

Tregs isolated from biopsy tissue were seeded into 48-well plates in 0.5 mL of Treg culture medium supplemented with 10% FBS (Capricorn Scientific, Ebsdorfergrund, Germany), 100 U/mL recombinant human IL-2 (Sci-Store, Moscow, Russia), and MACSiBead Particles pre-loaded with CD3 and CD28 antibodies (Treg Expansion Kit, Miltenyi Biotec, Bergisch Gladbach, Germany). The total number of cells was counted every 5 days, followed by replacement with fresh complete medium. The total cell counts were used to calculate the frequency (f) of cell divisions per day using the following equation: f = log_2_(Nd/N_0_), where Nd is the number of cells on a given cultivation day (d), and N_0_ is the initial cell number on day 0 (18, 19). Proliferating cells were photographed using a Biomed-3I inverted bright-field microscope (Biomed, Saint-Petersburg, Russia).

The study of Treg suppressive activity was performed by mixed lymphocyte reaction (MLR) according to previously described protocol (7, 8). Briefly, target CD4^+^CD25^–^ T cells were obtained from the same donor during immunomagnetic separation using the CD4+CD25+ Treg Isolation Kit (Miltenyi Biotec) as described above. The cells were recovered from the negative fraction generated during the isolation of CD4^+^CD25^+^ T lymphocytes. Target CD4^+^CD25^–^ cells were labeled with the vital fluorescent dye carboxyfluorescein succinimidyl ester (CFSE) using the CellTrace CFSE Cell Proliferation Kit (Thermo Fisher Scientific Inc., Waltham, MA). Peripheral blood mononuclear cells treated with mitomycin C (Kyowa Hakko Kirin, Tokyo, Japan) were used as feeder cells to stimulate the growth of target lymphocytes. The reaction was performed in 96-well V-bottom plates (Wuxi NEST Biotechnology, Jiangsu, China) in a final volume of 200 μL per well. The cells were cultured in RPMI-1640 medium supplemented with 10% FBS and 100 U/mL IL-2. Each reaction contained 1 × 10^4^ feeder cells. Tregs and target CD4^+^CD25^–^ T lymphocytes were mixed at ratios from ranging from 1: 1 to 1: 32. The total number of all cells per reaction was 2 × 10^5^. For the positive control, target CD4^+^CD25^−^ T lymphocytes were cultured in the presence of feeder cells but without Tregs. For the negative control, target CD4^+^CD25^–^ T lymphocytes were cultured in the absence of both feeder cells and Tregs. Each reaction was performed in triplicate. The cells were incubated for 5 days, and the number of labeled proliferating target cells was determined from the decrease in CFSE signal using flow cytometry.

### 2.7 Treg transfection with splice-switching oligonucleotides

Transfection of expanded salivary gland-derived Tregs was performed as we described in our previous work (7) using a 36-mer splice-switching oligonucleotides (SSO) capable to induce Δ7 FoxP3 mRNA splice variant (#Ins2 5’-atgtggcctgtccaggaggagtgcctgtaagtgggg-3’, and (#Del7 5’- ctgcaccgtgcagacctcctccctgccccccagcag-3’) with Lipofectamine 2000 (Invitrogen, Grand Island, NY). These oligonucleotides were designed to base-pair with splicing regulatory regions, thereby promoting the retention of exon 2 and the exclusion of exon 7 through competitive blocking of splicing regulatory protein binding to their sensitive cis-elements. Control cells were transfected with control non-specific 36-mer oligonucleotides (#Con1 5’-atgtgccgtaggtgaggcctcacgttcgttaaacgg-3’, and (#Con2 5’-gtgaggcctcacgttcgttaaacggatgtgccgtag-3’). All oligonucleotides were custom-synthesized by Evrogen (Moscow, Russia) and uniformly modified with 2’-O-(2-methoxy) ethyl sugars (2’MOE), a phosphorothioate backbone, and 5’-methyl cytosine. SSOs #Ins2 and #Con1 were conjugated with Cy5.5 fluorescent dye, whereas #Del7 and #Con2 were conjugated with Cy3 fluorescent dye. 5×10^4^ of Tregs from each patient were transfected and cultivated for 96 h as described above.

To determine the efficiency of transfection, Cy3-positive and Cy5.5-positive cells were counted by flow cytometry with a MACS Quant Analyzer 10 (Miltenyi Biotec, Bergisch Gladbach, Germany). The cellular load of the SSOs or control oligonucleotides was determined by mean fluorescence intensity (MFI) of Cy5.5-positive or Cy3-positive cells.

### 2.8 Statistics

Statistical differences between groups were analyzed using the Mann–Whitney U test in GraphPad Prism 8.0 (GraphPad Software, Inc., New York, NY, USA). For main comparisons (SjD vs HD, n = 20 per group), a p-value ≤ 0.05 was considered statistically significant. For subgroup analyses with small sample sizes (n = 3 per group), p-values are provided for descriptive purposes only; no formal statistical inference was made. Correction for multiple comparisons was not applied due to the exploratory nature of the study and the interdependence of the markers analyzed. All individual data points are shown in the figures to allow reader assessment.

## 3 Results

### 3.1 FoxP3 splice variants with the deletion of exon 7 are induced in peripheral blood Tregs from SjD patients

Four FoxP3 splice variants, resulting from pre-mRNA alternative splicing, are found in Tregs (**Figure 1A**). Using qPCR, we determined the mRNA levels of each FoxP3 splice variant in Tregs isolated from peripheral blood of SjD patients and HD. The proportion of the full-length (FL) FoxP3 splice variant was significantly decreased in Tregs from SjD patients (median 24.2%; **Figure 1B**) compared to HD (median 45.8%). The proportion of the Δ2 FoxP3 splice variant was also lower in Tregs of SjD patients (median 16.0%; **Figure 1D**) than in HD (median 47.0%). In contrast, the proportions of the Δ7 FoxP3 (median 38.5%; **Figure 1C**) and Δ2Δ7 FoxP3 (median 11.3%; **Figure 1E**) splice variants were significantly higher than those in Tregs from HD (medians 5.1% and 3.7%, respectively). The changes in the proportions of FoxP3 splice variants corresponded to changes in the proportion of Tregs in peripheral blood (**Figure 1F**). We observed a decrease in CD4^+^CD25^+^CD127^low^ Tregs in SjD patients (median 4.5%) compared to HD (median 8.2%). Individual data for the mRNA levels of FoxP3 splice variants in SjD patients and HD are shown in **Supplementary Figure 1** and **Supplementary Tables 1** and **2** in the **Supplementary File**.

**Figure 1:**
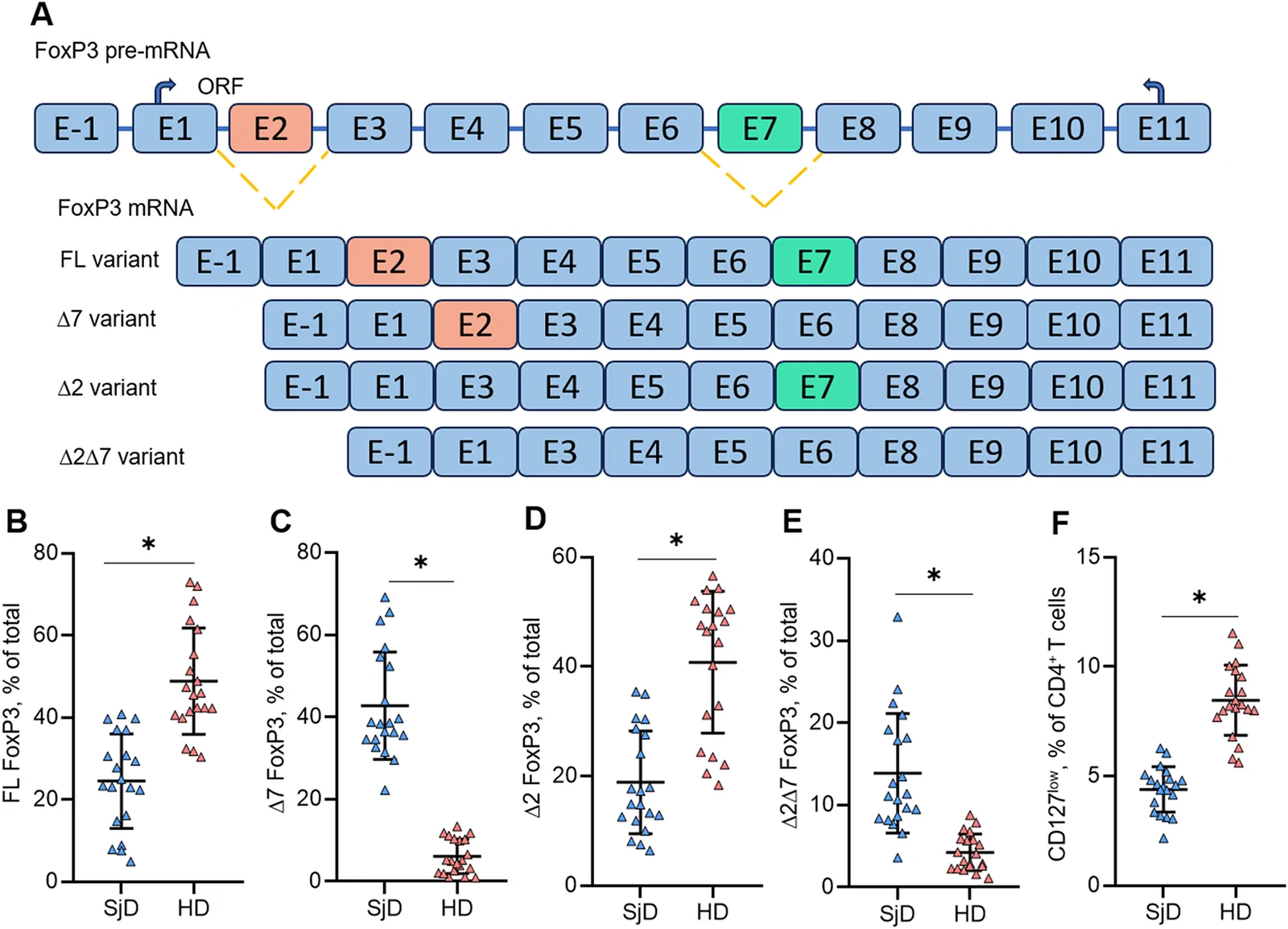
Increased expression of the Δ7 FoxP3 and Δ2Δ7 FoxP3 splice variants in Tregs is associated with a decreased proportion of these cells in the peripheral blood of patients with Sjögren’s disease (SjD). **(A)** Schematic representation of FoxP3 pre-mRNA alternative splicing. FoxP3 pre-mRNA consists of 12 exons and undergoes alternative splicing at exon 2 and/or exon 7, resulting in the formation of four splice variants. The open reading frame (ORF) is bounded by the start codon on exon 1 and the stop codon on exon 11. Proportions of **(B)** FL FoxP3, **(C)** Δ7 FoxP3, **(D)** Δ2 FoxP3, and **(E)** Δ2Δ7 FoxP3 mRNA in Tregs from SjD patients and healthy donors (HD) determined by real-time RT-PCR. **(F)** Proportion of CD4^+^CD25^+^CD127^low^ Tregs in the peripheral blood of SjD patients and HD determined by flow cytometry. Individual data points are shown; bars represent the mean ± standard deviation. * – p ≤ 0.05 vs. Tregs from HD by Mann-Whitney U test.

### 3.2 Predominance of Δ7 FoxP3 splice variant in Tregs isolated from biopsy material of SjD patients

Histological examination of the biopsy material revealed lymphocytic sialadenitis. Follicle-like structures with germinal centers were observed in the MSG tissue, representing a manifestation of extranodal lymphopoiesis (**Figure 2A**). We isolated Tregs from biopsy material of the salivary glands of SjD patients and determined the expression of FoxP3 splice variants. The proportion of Tregs within the CD4^+^ lymphocyte subpopulation from biopsies ranged from 3.12% to 6.38% (**Figures 2B, C**). The purity of isolated Tregs exceeded 90% (**Figure 2D**). mRNAs of all four FoxP3 splice variants were detected in Tregs from biopsies (**Figure 2E**; **Supplementary Figure 2** in the **Supplementary File**); however, the predominant splice variant was Δ7 FoxP3. The predominance of this splice variant in SjD patients’ biopsies corresponds to its elevated expression in peripheral blood Tregs.

**Figure 2:**
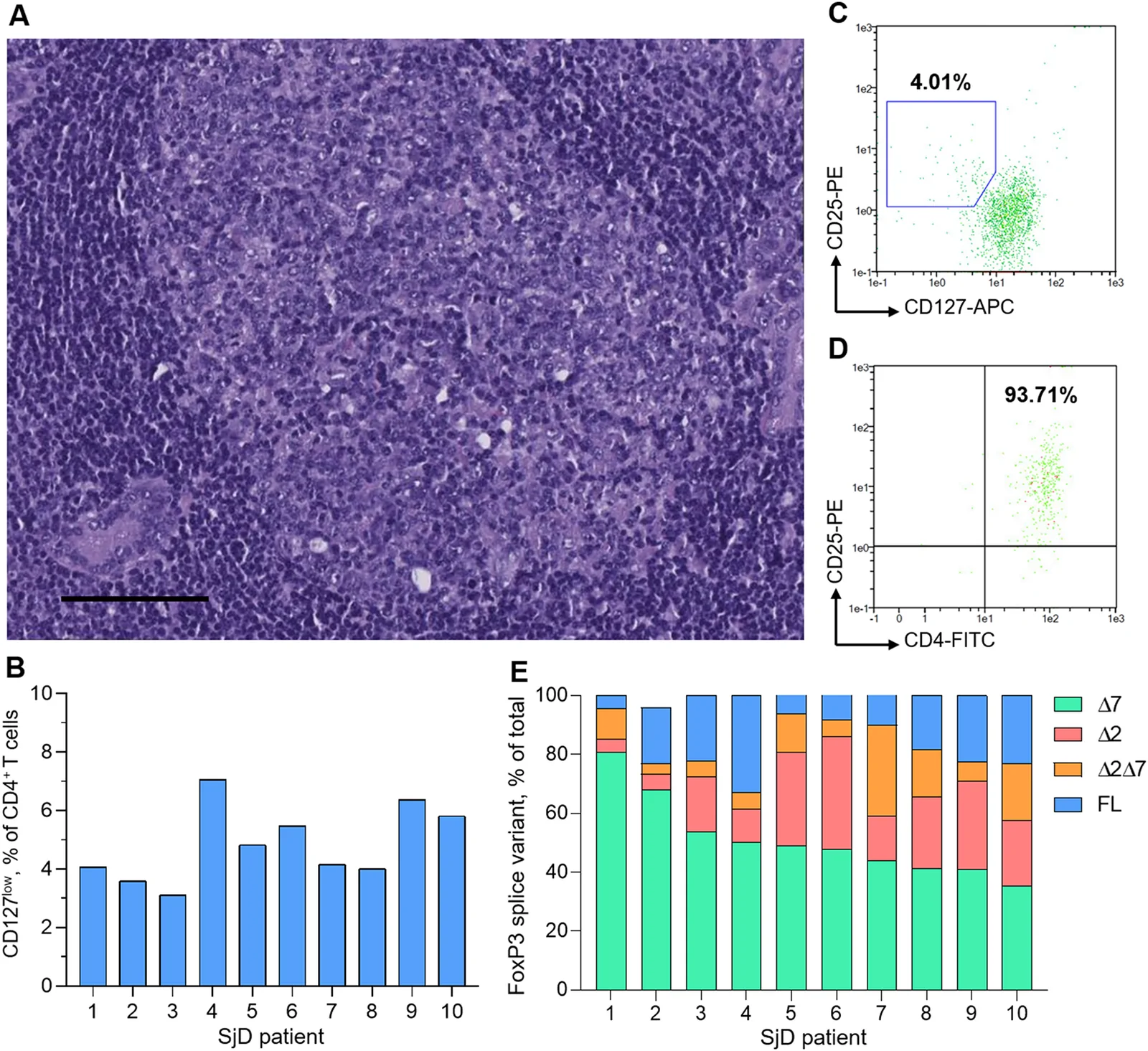
Increased expression of the Δ7 FoxP3 splice variant in Tregs isolated from biopsy material of ten SjD patients. **(A)** Hematoxylin and eosin staining of paraffin-embedded MSG biopsy from SjD patient. The parenchyma of the gland is replaced by a lymphocytic infiltrate with formation of follicle-like structures that contain germinal centers. Perpresentative photo is provided. Scale bars in the images correspond to 1000 µm. Magnification ×200. **(B)** Proportion of CD4^+^CD25^+^CD127^low^ Tregs within isolated CD4^+^ T cells determined by flow cytometry. Representative flow cytometry plots showing the gating strategy and the proportion of **(C)** CD25^+^CD127^low^ cells and **(D)** the purity of isolated CD4^+^CD25^+^ cells from biopsies. **(E)** Proportions of FoxP3 splice variant mRNAs in Tregs from SjD patients determined by qPCR. Patients are arranged in descending order of Δ7 FoxP3 mRNA proportion.

### 3.3 FoxP3 splice variants proportion remain unchanged during *ex vivo* proliferation

Very few Tregs can be isolated from biopsies. We selected three patients with relatively high (P1, P2, and P3) and three patients with relatively low (P8, P9, and P10) expression of the Δ7 FoxP3 splice variant and expanded these cells *ex vivo*. The proportions of FoxP3 splice variants were monitored every 5 days of proliferation by qPCR. We observed that expansion did not affect FoxP3 splice variant expression in Tregs with either high (**Figures 3A–C**) or low (**Figures 3D–F**) Δ7 FoxP3 levels. The Δ7 FoxP3 splice variant remained the predominant variant in the expanded cells.

**Figure 3:**
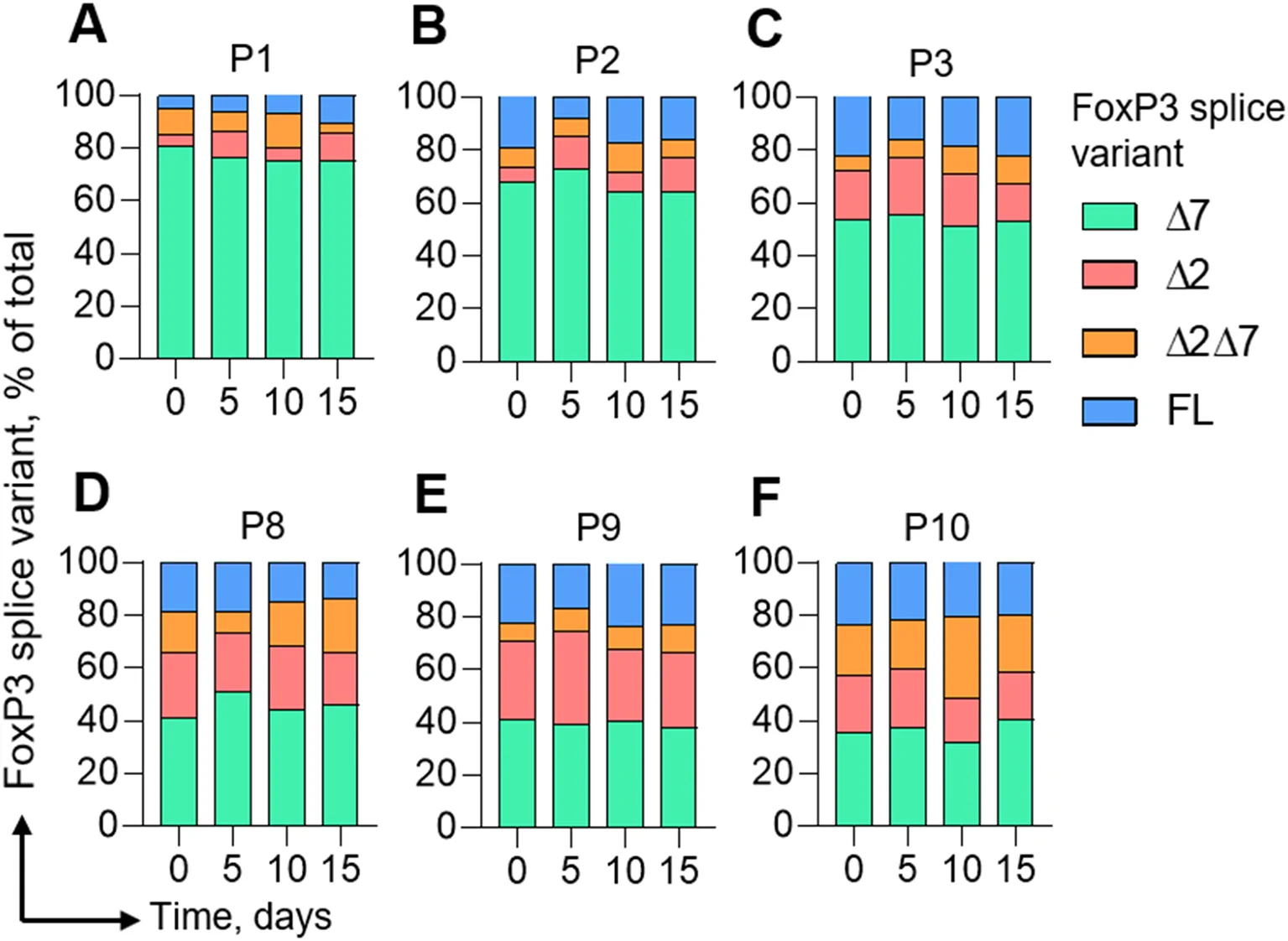
Proportion of FoxP3 splice variants mRNAs during ex vivo proliferation. mRNA levels of FoxP3 splice variants were determined by qPCR in Tregs from biopsy material of SjD patients with high **(A–C)** and low **(D–F)** expression of the Δ7 FoxP3 splice variant.

### 3.4 Tregs with high expression of Δ7 FoxP3 have reduced proliferative activity

We determined the total numbers of Tregs with high or low expression of Δ7 FoxP3 over 15 days of expansion. We observed that cells from patients with a high level of Δ7 FoxP3 proliferated less actively than those from patients with a low level of Δ7 FoxP3 (**Figure 4A**). By day 15 of proliferation, the total number of Tregs from patients P1–P3 was 7.3 ± 1.5 × 10^6^, with a mean frequency of cell cycle per day of 0.31, whereas the total number of Tregs from patients P8–P10 was 13.2 ± 2.4 × 10^6^, with a mean frequency of cell cycle per day of 0.47.

**Figure 4:**
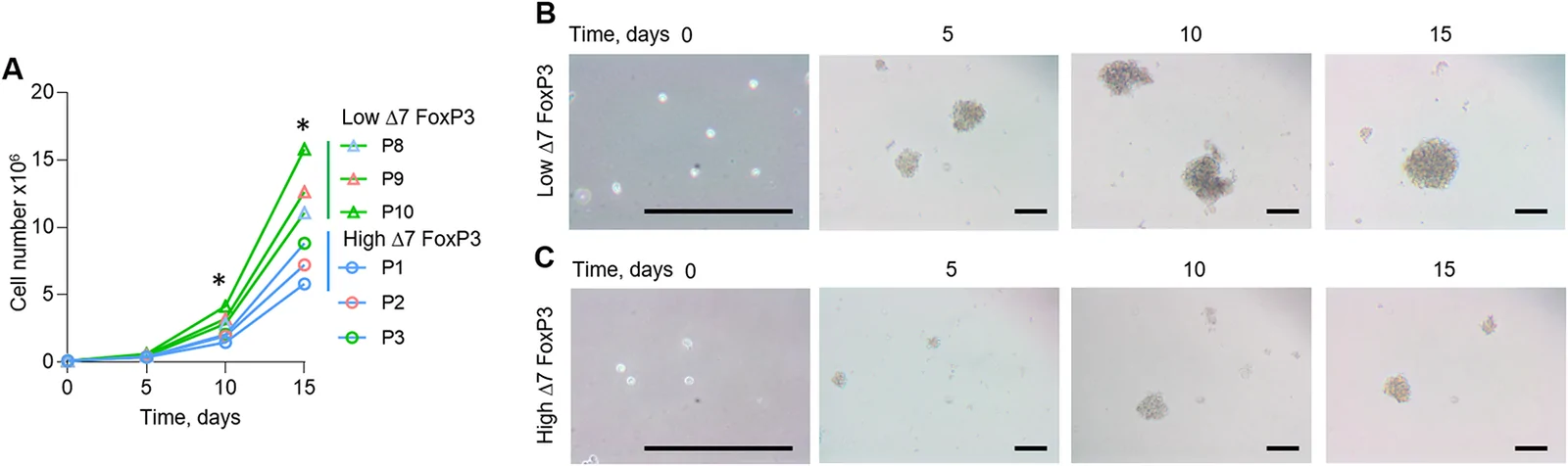
Reduced proliferative activity of Tregs with high Δ7 FoxP3. **(A)** Total cell numbers over 15 days of ex vivo proliferation of Tregs with low and high rate of Δ7 FoxP3 expression. Individual data are shown. * – p ≤ 0.05 compared to Tregs from patients with high Δ7 FoxP3 expression by Mann-Whitney U-test. Representative bright field microscopy images of proliferating Tregs with **(B)** low Δ7 FoxP3 expression or **(C)** high Δ7 FoxP3 expression. Bar scale 100 µm.

*In vitro* proliferation of Tregs is accompanied by the formation of proliferating clusters (20). We observed that Tregs with low Δ7 FoxP3 expression formed clusters by day 5 of proliferation (**Figure 4B**), while Tregs with high Δ7 FoxP3 expression only began to form them by day 10 (**Figure 4C**). By day 15 of proliferation, Tregs with high Δ7 FoxP3 formed fewer clusters, and their size was smaller.

The results of this experiment demonstrate that Tregs from biopsy material of patients with high Δ7 FoxP3 expression have reduced proliferative capacity.

### 3.5 Decreased expression of CD127^low^ and Helios by Tregs with elevated Δ7 FoxP3 splice variant

We determined the expression of molecules associated with the Treg phenotype by flow cytometry. No significant changes were observed in the expression of CD4, CD25, or CD152 markers (**Figures 5A–D, G, H**), and more than 90% of Tregs, both those with high and low Δ7 FoxP3 expression, were positive for these markers. In contrast, a significant decrease in CD127^low^ expression was observed in Tregs with high Δ7 FoxP3 (27.5 ± 6.7%) compared to those with low Δ7 FoxP3 (70.8 ± 2.3%). Helios expression was also significantly reduced in Tregs with high Δ7 FoxP3 (10.5 ± 7.6%) compared to those with low Δ7 FoxP3 (29.2 ± 6.5%). These results demonstrate that Tregs with high Δ7 FoxP3 expression have a reduced capacity to express CD127^low^ and Helios.

**Figure 5:**
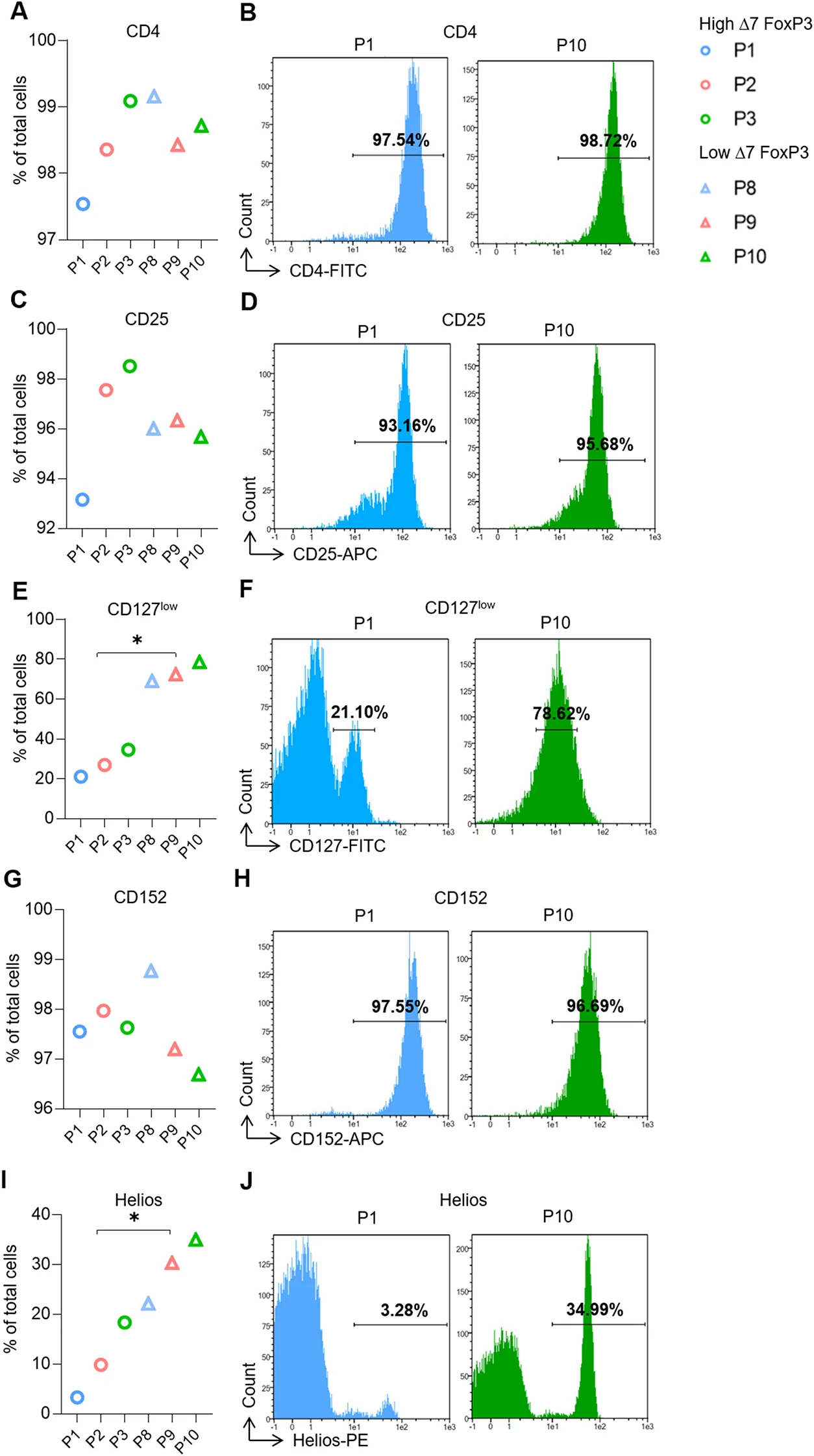
Immunophenotype of Tregs from SjD patients with low and high expression of the Δ7 FoxP3 splice variant. Proportion of cells expressing **(A)** CD4, **(C)** CD25, **(E)** CD127^low^, **(G)** CD152, and **(I)** Helios, as determined by flow cytometry. Individual data are shown. Representative flow cytometry plots showing **(B)** CD4, **(D)** CD25, **(F)** CD127^low^, **(H)** CD152, and **(J)** Helios expression in Tregs from SjD patients with low and high Δ7 FoxP3 expression. * – p ≤ 0.05 compared to Tregs from patients with low Δ7 FoxP3 expression by Mann-Whitney U-test.

### 3.6 Decreased suppressive activity of Tregs with high Δ7 FoxP3 expression

The suppressive activity of Tregs with high or low expression of Δ7 FoxP3 was studied in a mixed lymphocyte reaction. Tregs with high expression of Δ7 FoxP3 exhibited decreased suppressive activity. They were able to suppress autologous CD4^+^CD25^+^ T-lymphocytes only at a 1:4 ratio (**Figures 6A, B**), whereas Tregs with low Δ7 FoxP3 expression remained active even at a 1:16 ratio (**Figures 6C, D**). Individual proliferation data for target T cells from patients are shown in **Supplementary Figure 3** in the **Supplementary Data**.

**Figure 6:**
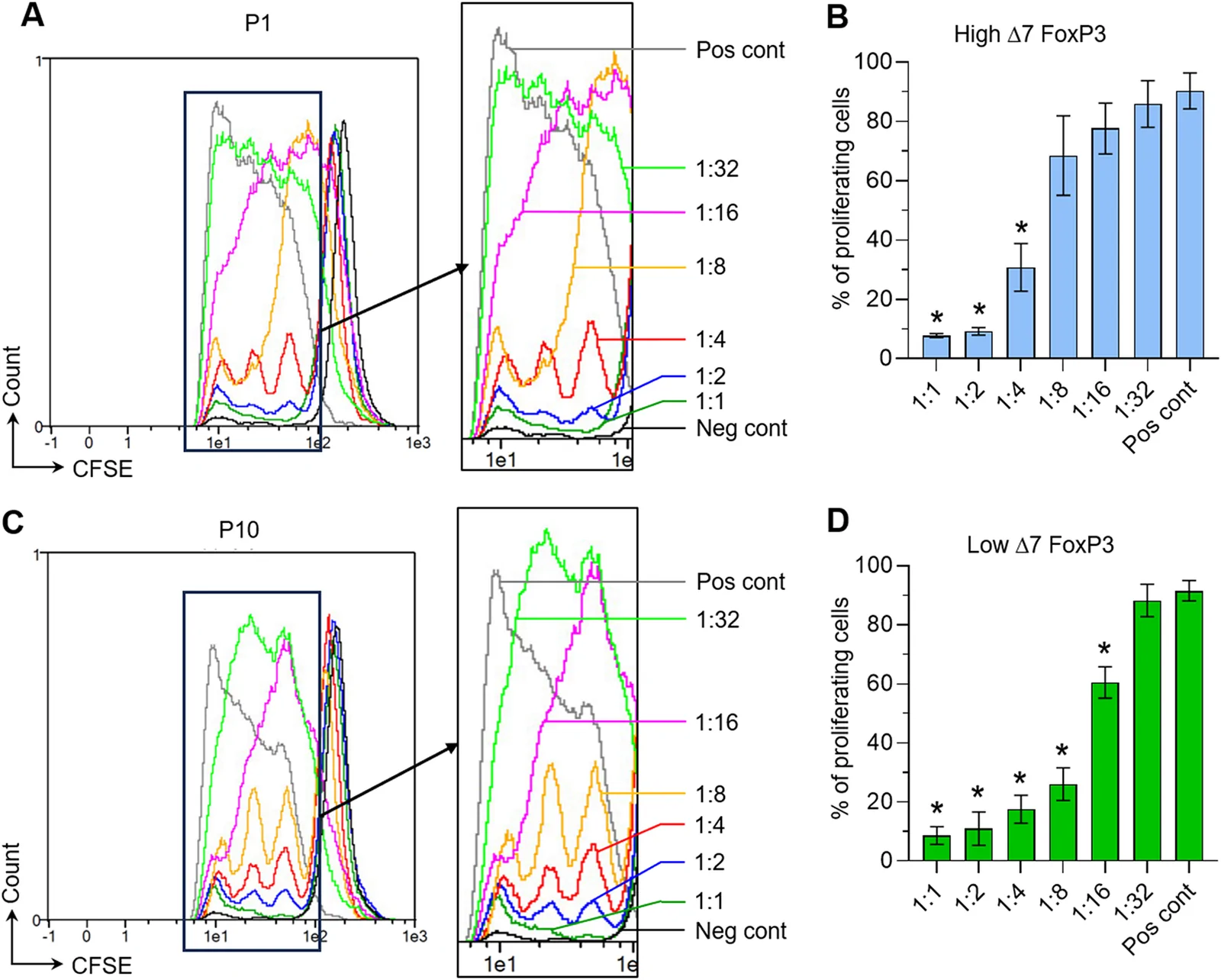
Suppressive activity of Tregs with high and low expression of the Δ7 FoxP3 splice variant. Tregs were incubated with FITC-labeled autologous CD4^+^CD25^−^ responder target cells in a range of ratios from 1:1 to 1:32 for five days in a mixed lymphocyte reaction (MLR), followed by flow cytometry counting of proliferated cells. Proliferative diagrams of target cells incubated with Tregs. Proliferative diagrams of target cells incubated with Tregs with **(A)** high expression of Δ7 FoxP3 and **(C)** low expression of Δ7 FoxP3. Proportion of proliferating target cells in MLR for Tregs with **(B)** high expression of Δ7 FoxP3 and **(D)** low expression of Δ7 FoxP3. Pos cont, positive control. Neg cont, negative control. The results are shown as mean ± standard deviation. N = 3. * – p ≤ 0.05 vs. positive control.

In order to explain the reduced suppressive activity of Tregs with elevated Δ7 FoxP3 expression, we assessed the expression of Treg-associated suppressive molecules by flow cytometry. The expression of the membrane molecule CD39 was significantly reduced in Tregs with high Δ7 FoxP3 expression, with only 5.4 ± 4.4% of cells being CD39-positive (**Figures 7A, B**) compared to 71.0 ± 8.6% of low Δ7 FoxP3 Tregs. Expression of another membrane molecule, CD223, was also reduced: only 11.6 ± 3.9% of high Δ7 FoxP3 Tregs were CD223-positive (**Figures 7C, D**) versus 68.9 ± 6.9% of low Δ7 FoxP3 Tregs. The expression of the proteases granzyme A (77.0 ± 2.7%; **Figures 7E, F**) and granzyme B (28.4 ± 6.0%; **Figures 7G, H**) was also reduced in Tregs with elevated Δ7 FoxP3 compared to those with low Δ7 FoxP3 (92.2 ± 0.4% and 40.2 ± 3.6%, respectively). In contrast, perforin expression did not differ significantly between high Δ7 FoxP3 (60.3 ± 17.0%) and low Δ7 FoxP3 (66.0 ± 12.0%) Tregs (**Figures 7I, J**).

**Figure 7:**
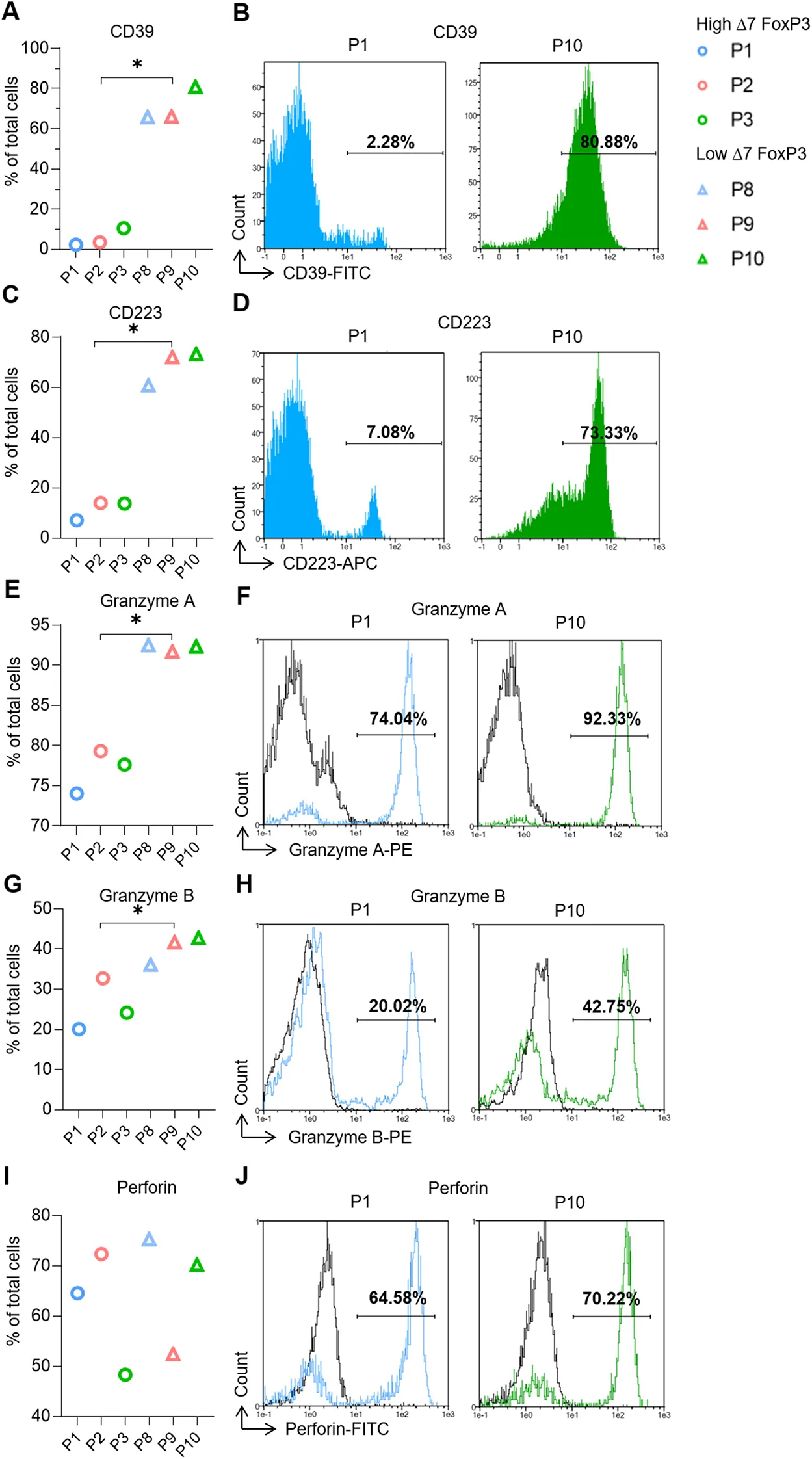
Expression of suppressive molecules by Tregs with high and low expression of Δ7 FoxP3 splice variant. Proportion of cells expressing **(A)** CD39, **(C)** CD223, **(E)** Granzyme A, **(G)** Granzyme B, and **(I)** Perforin determined by flow cytometry. Individual data are shown. Representative flow cytometry diagrams showing **(B)** CD39, **(D)** CD223, **(F)** Granzyme A, **(H)** Granzyme B, and **(J)** Perforin expression in Tregs from SjD patients with low and high expression of Δ7 FoxP3. Black lines indicate non-stimulated cells. * – p ≤ 0.05 compared to Tregs from patients with low Δ7 FoxP3 expression by Mann-Whitney U-test.

The ability of Tregs from biopsy material to produce suppressive cytokines was determined by measuring the concentrations of IL-10 and IL-35 in cell culture media after 15 days of cultivation using a Bio-Plex assay. The ability to produce IL-10 by Tregs with high Δ7 FoxP3 was decreased: 61.4 ± 24.5 pg/mL of this cytokine was detected in the media of high Δ7 FoxP3 Tregs compared to 153.0 ± 33.1 pg/mL in the media of Tregs with low Δ7 FoxP3 (**Figure 8A**). Tregs with high Δ7 FoxP3 also produced less IL-35 than those with low Δ7 FoxP3 (136.9 ± 49.9 pg/mL and 598.2 ± 96.7 pg/mL, respectively; **Figure 8B**).

**Figure 8:**
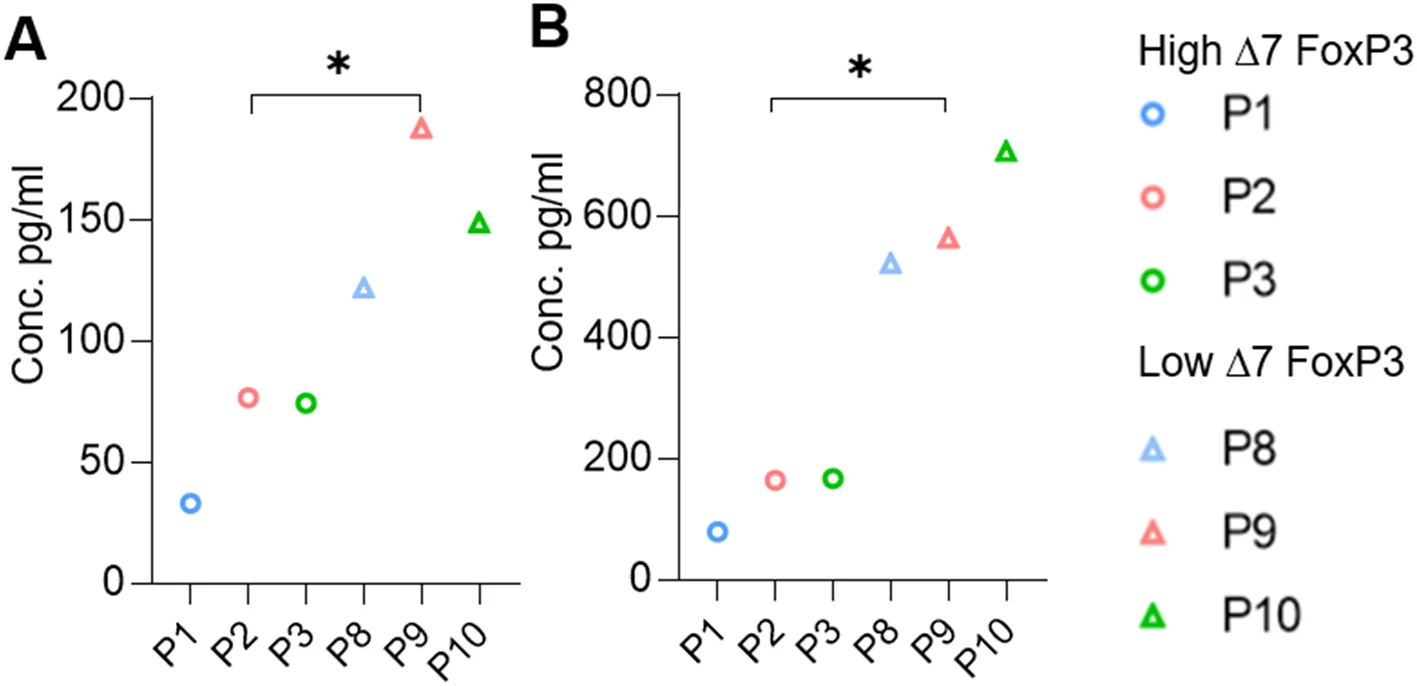
Ability to produce suppressive cytokines by Tregs with high and low expression of Δ7 FoxP3. Concentration of cytokines **(A)** IL-10 **(B)** IL-35 determined by Bioplex assay in growth media of SjD’s patients Tregs. Individual data are shown. * – p ≤ 0.05 compared to Tregs from patients with low Δ7 FoxP3 expression by Mann-Whitney U-test.

The results of this experiment demonstrate that Tregs with high expression of the Δ7 FoxP3 splice variant have reduced suppressive activity, which is associated with a decreased ability to synthesize suppressive molecules.

### 3.7 Immunophenotype and proliferation of salivary gland-derived Tregs with induced expression of Δ7 FoxP3 mRNA

To demonstrate that the changes in Treg-associated markers, molecules involved in the suppressive activity, or proliferative activity of Tregs are directly attributed to Δ7 FoxP3, and are not the result of remote implications of SjD, we induced the expression of Δ7 FoxP3 mRNA in expanded salivary gland-derived Tregs from SjD patients using SSOs. Transfection efficiency was monitored every 24 hours for 96 hours post-transfection and was not lower than 66.2% at the end of the experiment (**Supplementary Figure 4** in the **Supplementary File**).

Transfection of Tregs with SSO #Ins2, targeting exon 2 retention, and SSO #Del7, targeting exon 7 exclusion, induced predominant (96.6 – 98.2%) expression of Δ7 FoxP3 at 96 h post-transfection. The expression of other FoxP3 splice variants did not exceed 2%. Tregs with induced Δ7 FoxP3 expression showed no changes in the expression of surface markers CD4, CD25, or CD152 compared to control cells (**Figure 9B**). However, the expression of CD127^low^ marker decreased more than twofold, while the expression of Helios decreased up to fivefold following Δ7 FoxP3 induction. After Δ7 FoxP3 induction, we also observed a significant decrease in the expression of molecules involved in the suppressive functions of Tregs. The proportion of cells expressing CD39 decreased nearly tenfold, CD223 decreased fourfold, and granzyme A decreased by half (**Figure 9C**).

**Figure 9:**
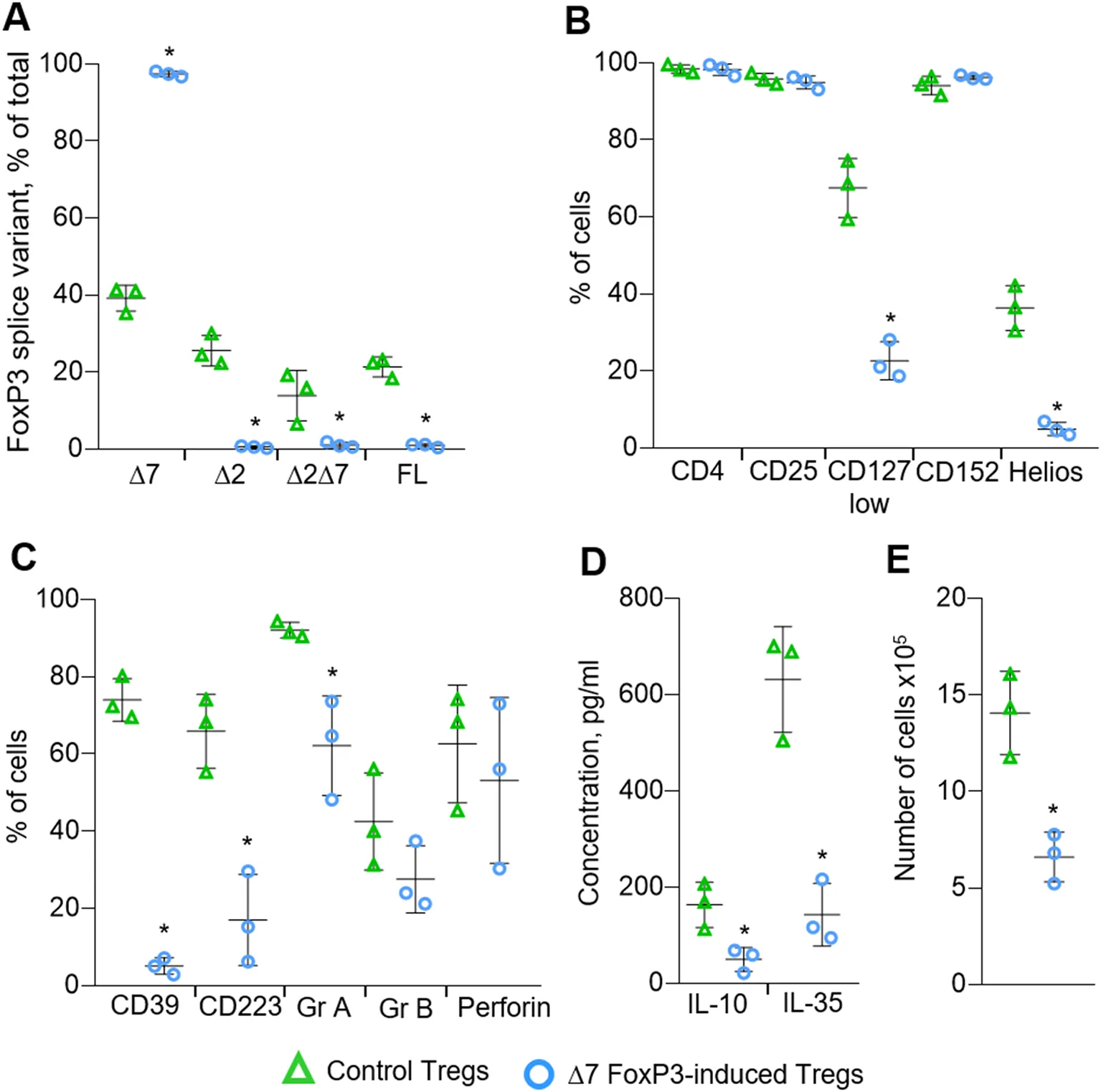
Cell markers, cytokines, and proliferation of Tregs with induced expression of Δ7 FoxP3 mRNA. Tregs were isolated from major salivary gland biopsies, expanded ex vivo, and transfected with SSOs to selectively induce the expression of Δ7 FoxP3. **(A)** Proportion of FoxP3 mRNA splice variants in control Tregs (transfected with control non-specific oligonucleotides) or in Tregs transfected with Δ7 FoxP3-inducing SSOs determined by real-time RT-PCR. **(B)** Percentage of cells expressing Treg-associated markers CD4, CD25, CD127^Low^, CD152, and Helios determined by flow cytometry. **(C)** Percentage of cells expressing molecules involved in Treg-mediated suppression: CD39, CD223, granzyme A (Gr A), granzyme B (Gr B), and perforin determined by flow cytometry. **(D)** Concentration of suppressive cytokines in the Treg culture media determined by Bio-Plex assay. **(E)** Total cell number after 96 h of culturing ex vivo posttransfection. Data from each of the three SjD patients are shown individually and presented as mean ± SD; *p ≤ 0.05 compared to control Tregs.

The proportion of cells expressing granzyme B and perforin did not differ from that of control cells. Tregs with induced Δ7 FoxP3 demonstrated a reduced potential to produce suppressive cytokines. The concentrations of IL-10 and IL-35 were significantly decreased in Δ7 FoxP3-expressing cells (**Figure 9D**). The induction of Δ7 FoxP3 also affected cell proliferation. At 96 hours post-transfection, the total number of proliferated Δ7 FoxP3-induced Tregs was almost two times lower than that of control cells (**Figure 9E**).

The results of this experiment were in good agreement with the data obtained in non-transfected MSG-derived expanded Tregs and demonstrated that the changes in Treg-associated surface markers, suppressive molecules, and proliferative intensity are directly attributed to elevated expression of Δ7 FoxP3 in SjD patient Tregs.

## 4 Discussion

Although most studies on Treg function have been performed on cells isolated from peripheral blood, considerable attention has been directed toward Tregs in lymph nodes, organs, and tissues (21–25). Migration of Tregs from the peripheral blood allows these cells to reach sites where immune inflammation develops. To regulate specific T helper (Th)1-, Th2-, or Th17-type immune responses, the phenotype and function of Tregs change in peripheral organs, leading to the co-expression of IRF4, T-bet, and STAT-3 (26–29).

Lymphocytic infiltration in SjD may lead to the formation of ectopic structures resembling germinal centers (GCs) in peripheral lymphoid tissue. According to previously reported data, 25.1 ± 5.0% of patients with SjD have ectopic GCs in their salivary glands (30). Structurally, GCs are formed by antigen-specific T and B lymphocytes within a network of follicular dendritic cells and activated endothelial cells. The cytokine profile of GCs reveals increased concentrations of pro-inflammatory Th17-associated cytokines, including IL-17, IL-1β, and the IL-23 subunit IL-12p40 (31). Such a milieu provides an environment favorable for B-cell proliferation, somatic hypermutation, and immunoglobulin class switching, making GCs a focus of *in situ* autoantibody production. Consequently, the formation of ectopic GCs in SjD is associated with a higher risk of lymphoma development (32).

Aberrant homing of infiltrating lymphocytes is directed by a complex network of chemokines. Recent findings have elucidated the spatiotemporal dynamics of this process, demonstrating that C-C chemokine receptor 1 (CCR1) and C-C ligand 5 (CCL5) are involved in the early stages of salivary gland infiltration. Specifically, CCR1^hi^/CCL5^hi^ macrophages are among the first cells to infiltrate the salivary glands in SjD (33). They subsequently recruit CCL5^hi^ T cells through chemokine signaling, which, in turn, recruit and activate B cells, driving the formation of lymphocytic infiltrates and glandular destruction. Beyond this CCR1/CCL5 axis, the C-X-C motif chemokine ligand 13 (CXCL13)/C-X-C motif chemokine receptor 5 (CXCR5) pathway is critical for the organization of ectopic lymphoid structures. CXCL13 is significantly elevated in the serum and salivary glands of SjD patients and correlates with the degree of lymphocytic infiltration, ectopic lymphoid structure formation, and lymphoma risk (34). This chemokine milieu is crucial for the recruitment and positioning of specific T-cell subsets. Tregs within the GC structure, termed follicular Tregs (Tfr), are characterized by high expression of CXCR5, which directs their migration in response to the CXCL13 gradient. These cells also express programmed cell death 1 (PD-1), inducible T-cell costimulator (ICOS), and the transcription factor B cell lymphoma 6 (Bcl-6) (35). Importantly, specific homing of Tfr is supported by the expression of nuclear factor of activated T cells 2 (NFAT2) and Bcl-6, which upregulate CXCR5 expression (36). Interestingly, some studies indicate that Tfr within GCs may lose FoxP3 expression and suppressive capacity; however, it should be noted that these observations were made in mice (37). The role of Tfr is to restrict GC reactions, either indirectly through effects on follicular Th (Tfh) cells or directly through effects on B cells, thereby limiting autoimmune responses (35). Tfh cells release IL-21 and IL-4, contributing to B-cell differentiation and proliferation. Tfr suppress Tfh proliferation and their production of IL-4 and IL-21. In the salivary glands of patients with SjD, the number of Tfh cells is increased, which positively correlates with the ESSDAI (38). The frequency of CXCR5^+^ Tfr in salivary glands has also been reported to be increased compared to healthy individuals (39). In the blood of SjD patients, a subset of circulating Tfr (cTfr) has been identified, with a frequency higher than in healthy donors. It was demonstrated that cTfr cells are immature and not fully specialized in suppressing humoral responses (40). They are phenotypically distinct from Tfr and have a naïve-like phenotype, as they do not express ICOS, PD-1, Bcl-6, or CD45RO, and express high levels of CD45RA and low levels of FoxP3. The study by Fonseca et al. revealed that acquisition of a CXCR5^+^FoxP3^+^ phenotype in tissues precedes access to the GC, where the cells acquire a fully mature phenotype (40).

There are limited data available on the mechanisms of Tfr suppressive function in humans. In contrast to peripheral Tregs that are not resident in GCs, IL-2 deprivation by Tfr may not contribute to Tfh suppression. This is explained by the fact that Tfh differentiation is directly inhibited by IL-2 (41). Moreover, CD25 expression by Tfr may be lower than that of peripheral Tregs. Authors have described the existence of a CD25^−^/^low^ Tfr population within GCs (42). They proposed that downregulation of CD25 reflects the maturational stage of GC-localized Tfr, with the CD25^−^/^low^ phenotype considered mature. It was hypothesized that downregulation of CD25 insulates Tfr from the IL-2-dependent differentiation pathway and directs them toward an IL-2-antagonized pathway defined by Bcl-6 and characterized by CXCR5, TIGIT, ICOS, B- and T-lymphocyte attenuator (BTLA), and PD-1, which are also associated with the Tfh phenotype. Intriguingly, CD25^−^/^low^ Tfr represent a FoxP3-expressing suppressive Treg population, although their suppressive activity was slightly reduced compared to CD25^+^ Tfr. Many IL-2-dependent genes, such as Klrg1, Itgae (CD103), Prdm (BLIMP-1), Il10, and Gzmb (granzyme B), were downregulated, whereas expression of GITR, Helios, and CTLA-4 was retained. The existence of the CD25^−^/^low^ Tfr population reflects the plasticity of Tregs, as this subset acquires a transcriptional signature that is mixed between Tfh and Treg cells while retaining suppressive function. At the same time, it should be noted that the presence of this population and aspects of its molecular and functional characteristics in SjD remain to be elucidated. It also remains unclear how Tfr suppress B cells within GCs. Co-culture experiments have shown that Tfr inhibit the expression of a subset of effector genes and multiple metabolic pathways in B cells, including glycolysis, glutaminolysis, one-carbon metabolism, serine biosynthesis, purine biosynthesis, and their upstream mediators, Myc and mTOR (43). Notably, these results were obtained in mice. The impact of Tfr dysregulation in SjD warrants further investigation; however, one study using a mouse model of SjD showed that ablation of Bcl6 in Tregs greatly enhanced disease development (44).

Our results demonstrate an association of reduced suppressive and proliferative activity of Tregs derived from MSGs of SjD patients with high expression of Δ7 FoxP3 splice variant (**Figures 1**–**3**) in these cells (**Figure 4**). As described above, Tfr within GCs may downregulate suppressive activity, however not a single study has addressed the profile of FoxP3 splice variants in this cellular population. We hypothesize that the observed dysfunction may be due to the induction of Δ7 FoxP3 splice variant. Moreover, among individuals with increased Δ7 splice variant expression those with the highest and lowest levels of Δ7 variant has been selected for further analysis. Differences in marker expression have been revealed (**Figure 5**): Tregs with high level of Δ7 splice variant downregulated expression of Helios and CD127^low^, compared to Tregs with low level of Δ7 variant. Expression of CD4 and CD25 was not different among two groups and remained high. Ability to express suppressive molecules has also been analyzed. Tregs with high level of Δ7 downregulated expression of CD39, CD223 and granzymes A and B (**Figures 6**–**8**). Notably, no difference was observed in CD152 and perforin expression. Importantly, Tregs from MSGs of patients were identified as CD4^+^CD25^+^CD127^low^ cells. The results obtained on the expanded MSG-isolated Tregs were in good agreement with the results of functional experiments. The induction of Δ7 FoxP3 with specific SSOs resulted in a decrease in cells expressing CD127low, CD152, and Helios cell markers (**Figure 9**), as well as a decrease in the expression of suppressive molecules CD39, CD223, and perforin. Such cells were also less potent in the synthesis of suppressive cytokines IL-10 and IL-35 and demonstrated decreased proliferative potential.

An intriguing question remains regarding the biological role of FoxP3 alternative splicing. Since the pathogenesis of SjD is not fully understood, it is unclear whether alterations in this process represent a cause or a consequence of the disease. One hypotetical possibility is that decreased Treg activity resulting from altered FoxP3 alternative splicing may represent a mechanism by which the organism fine-tunes the balance of the immune response. This is consistent with the finding that GC formation in SjD is associated with a high risk of lymphoma development (32). Given that Tregs are generally considered poor prognostic markers in cancer due to their inhibition of antitumor immunity, a reduction in their function resulting from altered FoxP3 alternative splicing might potentially reflect an attempt by the organism to alleviate the blockade of antitumor responses. From this perspective, we propose that regulation of FoxP3 alternative splicing represents a novel intrinsic mechanism through which Tregs may control immune equilibrium. This interpretation remains hypothetical and requires gaining a more precise understanding of whether the mature CD25^−^/^low^ Tfr population described in the literature (42) could be linked to, or serve as a predisposing factor for lymphomagenesis under disease conditions.

Several limitations of the study must be denoted. First, the sample size, particularly for MSG biopsies (n = 10), is relatively small, and the subdivision into high- and low-Δ7 FoxP3 expression groups further reduced the number of patients to three per group. Consequently, the comparisons of marker expression, proliferative activity, and suppressive function between these subgroups are underpowered and should be interpreted as descriptive rather than confirmatory. The consistency of the observed trends across multiple markers, however, supports the biological plausibility of our findings. The **Supplementary Table 3** in the **Supplementary File** demonstrates all individual marker values for each donor to ensure full transparency and facilitate reader assessment. Second, the clinical heterogeneity of the patient cohort, including varied disease duration, disease activity, and previous treatments, may represent a confounding factor. Although all treated patients had discontinued therapy at least two years prior to enrollment, and the same splicing profile was observed across therapy-naïve and previously treated patients, we cannot exclude the possibility of long-term effects. Third, we did not assess CXCR5 expression on MSG-derived Tregs. This marker is critical for identifying the Tfr subset that localizes to GCs. Without it, we cannot definitively determine whether the analyzed Treg population represents bona fide Tfr cells, other MSG-infiltrating Treg subsets, or a mixed population. While we suggest that the observed cells might represent a transitional state with reduced suppressive function that has not yet downregulated CD25, this remains speculative and requires further investigation with CXCR5 staining and phenotyping of Treg subsets in MSG infiltrates. Future work with larger, well-characterized cohorts is needed to validate these observations and to define how MSG Treg phenotype, function, and FoxP3 splicing correlate with defined clinical stages, disease activity, and lymphoma predisposition in SjD. Although we demonstrated the presence of GCs within MSG biopsies, we did not validate the presence of Tregs in them immunohistochemically. That is why it may be possible that the analyzed Treg population may be part of the lymphocytic infiltrate of the MSG, not necessarily containing GCs in its structure. However, alternative splicing of FoxP3 has not been described in any articles devoted to non-GC MSG-infiltrating Tregs.

In conclusion, our findings demonstrate an upregulation of the FoxP3 Δ7 splice variant in MSG Tregs from SjD patients. Tregs with high Δ7 expression showed impaired proliferation and suppression, linked to reduced suppressive markers and cytokines. These results underscore the functional impact of Treg-associated FoxP3 splicing dysregulation in SjD.

## Data Availability

The original contributions presented in the study are included in the article/**Supplementary Material**. Further inquiries can be directed to the corresponding author.
