# Supplementary material for: Association of phenotypic and functional characteristics of regulatory T cells from minor salivary glands with expression of the delta 7 FoxP3 splice variant in patients with Sjögren’s disease
Source: Front Immunol. 2026 Oct 2;17:1959200. doi: 10.3389/fimmu.2026.1959200 (PMC13672494; doi:10.3389/fimmu.2026.1959200)
Supplement: Supplementary file 1 [file DataSheet1.docx]

Supplementary Material

Association of Phenotypic and Functional Characteristics of Regulatory T Cells from Minor Salivary Glands with Expression of the delta 7 FoxP3 Splice Variant in Patients with Sjögren's Disease

Varvara G. Blinova^1^, Yulia A. Gladilina^1^, Ekaterina B. Rоdionova^2^, Vladimir I. Vasilyev^3^, Natalia S. Shornikova^3^, Anastasiya N. Shishparenok^1^ and Dmitry D. Zhdanov^1,4*^

^1^Laboratory of Medical Biotechnology, Institute of Biomedical Chemistry, Moscow, Russia

^2^DDM Clinic, Moscow, Russia

^3^Dr. Laskov's Clinic, Moscow, Russia

^4^Department of Biochemistry, Peoples’ Friendship University of Russia named after Patrice Lumumba (RUDN University), Moscow, Russia

*** Correspondence:**
Dmitry D. Zhdanov
Institute of Biomedical Chemistry, Pogodinskaya St. 10/8, Moscow, 119121, Russia

E-mail address: zhdanovdd@gmail.com (D.D. Zhdanov).

**Supplementary Figure 1.** Expression of FoxP3 splice variants. mRNA levels of each FoxP3 splice variant in peripheral blood Tregs from (A) patients with Sjögren's disease (SjD) and (B) healthy donors (HD). Patients and donors are ranked by FL FoxP3 expression level.

**Supplementary Table 1****.** Individual data on the proportions of FoxP3 splice variants, total FoxP3 expression in Tregs, and the proportion of Tregs in the peripheral blood of patients with Sjögren's disease.

| SjD patient | FL variant, % of total splice variants | ∆2 variant, % of total splice variants | ∆7 variant, % of total splice variants | ∆2∆7 variant, % of total splice variants | Total FoxP3 expression | Treg, % of CD4^+^ |
| --- | --- | --- | --- | --- | --- | --- |
| 1 | 40.8 | 24.1 | 31.5 | 3.6 | 0.435 | 6.07 |
| 2 | 39.8 | 12.6 | 36.5 | 11.1 | 0.443 | 4.83 |
| 3 | 39.7 | 11.9 | 39.7 | 8.7 | 0.455 | 5.47 |
| 4 | 37.1 | 12.9 | 38.6 | 11.4 | 0.386 | 4.16 |
| 5 | 37.0 | 14.9 | 38.4 | 9.7 | 0.488 | 4.51 |
| 6 | 30.8 | 14.8 | 36.3 | 18.2 | 0.528 | 4.60 |
| 7 | 30.6 | 30.4 | 29.6 | 9.5 | 0.370 | 3.12 |
| 8 | 29.4 | 27.6 | 34.6 | 8.4 | 0.395 | 4.38 |
| 9 | 27.8 | 28.7 | 32.7 | 10.7 | 0.386 | 4.81 |
| 10 | 25.0 | 18.0 | 34.6 | 22.4 | 0.244 | 3.06 |
| 11 | 23.4 | 8.1 | 35.6 | 32.9 | 0.332 | 3.20 |
| 12 | 23.1 | 35.4 | 22.3 | 19.2 | 0.399 | 3.81 |
| 13 | 23.0 | 30.6 | 38.8 | 7.7 | 0.211 | 6.28 |
| 14 | 22.4 | 7.5 | 63.5 | 6.6 | 0.503 | 4.64 |
| 15 | 16.2 | 10.0 | 65.6 | 8.2 | 0.3919 | 5.20 |
| 16 | 14.8 | 6.4 | 54.7 | 24.1 | 0.452 | 4.88 |
| 17 | 8.9 | 17.7 | 52.4 | 21.0 | 0.391 | 5.07 |
| 18 | 7.9 | 17.3 | 56.9 | 17.9 | 0.358 | 3.36 |
| 19 | 7.6 | 35.0 | 43.9 | 13.5 | 0.299 | 4.39 |
| 20 | 4.9 | 13.3 | 69.2 | 12.7 | 0.327 | 2.17 |

FL, full-length variant; SjD, Sjögren's disease; ∆ denotes the number of deleted exons. Patients are ordered by decreasing FL FoxP3 splice variant proportion.

**Supplementary Table 2.** Individual data on the proportions of FoxP3 splice variants, total FoxP3 expression in Tregs, and the proportion of Tregs in the peripheral blood of healthy donors.

| HD | FL variant, % of total splice variants | ∆2 variant, % of total splice variants | ∆7 variant, % of total splice variants | ∆2∆7 variant, % of total splice variants | Total FoxP3 expression | Treg, % of CD4^+^ |
| --- | --- | --- | --- | --- | --- | --- |
| 1 | 73.0 | 22.1 | 2.1 | 2.9 | 0.626 | 9.5 |
| 2 | 72.0 | 18.4 | 5.2 | 4.3 | 0.473 | 5.8 |
| 3 | 68.4 | 20.5 | 3.2 | 7.9 | 0.751 | 6.3 |
| 4 | 63.7 | 24.4 | 5.0 | 6.8 | 0.638 | 7.7 |
| 5 | 61.5 | 23.5 | 11.8 | 3.2 | 0.475 | 11.5 |
| 6 | 51.4 | 46.5 | 1.1 | 1.1 | 0.722 | 8.0 |
| 7 | 55.4 | 32.9 | 6.6 | 5.2 | 0.601 | 6.8 |
| 8 | 47.4 | 48.3 | 2.7 | 1.6 | 0.654 | 8.8 |
| 9 | 46.0 | 50.6 | 1.1 | 2.3 | 0.411 | 10.0 |
| 10 | 45.5 | 47.6 | 3.9 | 2.9 | 0.451 | 5.6 |
| 11 | 48.9 | 31.2 | 11.2 | 8.8 | 0.49 | 8.5 |
| 12 | 42.4 | 50.0 | 5.3 | 2.3 | 0.42 | 8.1 |
| 13 | 42.4 | 47.5 | 4.4 | 5.8 | 0.697 | 8.2 |
| 14 | 42.3 | 54.3 | 0.9 | 2.6 | 0.388 | 9.8 |
| 15 | 41.5 | 44.5 | 11.8 | 2.1 | 0.450 | 11.1 |
| 16 | 40.6 | 40.3 | 13.4 | 5.7 | 0.801 | 10.2 |
| 17 | 39.9 | 54.0 | 1.9 | 4.1 | 0.435 | 8.3 |
| 18 | 32.4 | 50.5 | 10.0 | 7.1 | 0.636 | 8.0 |
| 19 | 31.8 | 52.0 | 10.3 | 5.9 | 0.399 | 8.9 |
| 20 | 30.4 | 56.6 | 10.4 | 2.6 | 0.415 | 8.1 |

FL, full-length variant; HD, healthy donor; ∆ indicates the number of deleted exons. The donors are ranged by the decrease of FL FoxP3 splice variant proportion.

**Supplementary Figure 2.** Proportions of FoxP3 splice variant mRNAs in Tregs from biopsies of patients with Sjögren's disease.

**Supplementary Figure 3**. Suppressive activity of Tregs from biopsy material of patients with Sjögren's disease. Tregs were incubated with FITC-labeled autologous CD4^+^CD25^−^ responder target cells in a range of ratios from 1:1 to 1:32 for five days in a mixed lymphocyte reaction, followed by flow cytometry counting of proliferated cells. Individual proliferation data of target CD4⁺CD25⁻ T cells from patients with (A) high and (B) low expression of the ∆7 FoxP3 splice variant. Pos cont, positive control.

**Supplementary Figure 4**. Transfection efficiency of salivary gland-derived Tregs with SSOs. Representative flow cytometry plots of cells transfected with Cy3- or Cy5.5-labeled oligonucleotides within 96 h posttransfection: (A) nontransfected cells; (B) cells transfected with control nonspecific oligonucleotide #Con1 and #Con2; (C) cells transfected with oligonucleotide #Ins2, which is capable of inducing insertion of exon 2 and oligonucleotide #Del7, which is capable of inducing deletion of exon 7. (D, E,) Transfection efficiency. (F,G) Cellular load of the SSOs or control oligonucleotides determined by mean fluorescence intensity (MFI). The results are shown as the mean ± SD. N= 3. *p ≤ 0.05 vs. cells at the 0 h time point by the Mann–Whitney U-test.

**Supplementary Table 3.** Individual values of phenotypic markers, suppressive molecules, and cytokines in Tregs isolated from minor salivary glands of SjD patients with high (P1–P3) and low (P8–P10) expression of the ∆7 FoxP3 splice variant. Data are shown as percentages (for flow cytometry markers) or pg/mL (for cytokines). For each group, mean ± standard deviation is also provided. All values represent single measurements per donor.

| **Marker** | **High ∆7 FoxP3 (P1–P3)** | | | **Mean ± SD** | **Low ∆7 FoxP3 (P8–P10)** | | | **Mean ± SD** |
| --- | --- | --- | --- | --- | --- | --- | --- | --- |
|  | **P1** | **P2** | **P3** | **High ∆7** | **P8** | **P9** | **P10** | **Low ∆7** |
| *Phenotype* | | | | | | | | |
| CD4, % | 97.54 | 96.36 | 99.09 | 97.66 ± 1.38 | 99.16 | 99.43 | 98.72 | 99.10 ± 0.36 |
| CD25, % | 93.16 | 97.56 | 98.52 | 96.41 ± 2.85 | 96.01 | 96.34 | 95.68 | 96.01 ± 0.33 |
| CD127^low^, % | 21.1 | 26.94 | 34.56 | 27.53 ± 6.77 | 69.18 | 72.44 | 78.6 | 73.41 ± 4.75 |
| Helios, % | 3.28 | 9.85 | 18.35 | 10.49 ± 7.56 | 22.15 | 30.33 | 34.99 | 29.16 ± 6.48 |
| CD152, % | 97.55 | 97.97 | 97.63 | 97.72 ± 0.22 | 98.77 | 97.2 | 96.69 | 97.55 ± 1.08 |
| *Suppressive molecules* | | | | | | | | |
| CD39, % | 2.28 | 3.53 | 10.44 | 5.42 ± 4.40 | 65.85 | 66.17 | 80.88 | 70.97 ± 8.55 |
| CD223, % | 7.08 | 13.99 | 13.74 | 11.60 ± 3.90 | 60.8 | 72.04 | 73.33 | 68.72 ± 6.90 |
| Granzyme A, % | 74.04 | 79.32 | 77.65 | 77.00 ± 2.67 | 92.51 | 91.7 | 92.33 | 92.18 ± 0.41 |
| Granzyme B, % | 20.02 | 32.72 | 24.16 | 25.63 ± 6.47 | 36.12 | 41.67 | 42.75 | 40.18 ± 3.55 |
| Perforin, % | 64.58 | 72.33 | 48.32 | 61.74 ± 12.15 | 75.34 | 52.47 | 70.22 | 66.01 ± 12.02 |
| *Cytokines* | | | | | | | | |
| IL-35, pg/mL | 79.25 | 164.04 | 167.37 | 136.89 ± 49.89 | 522.74 | 564.53 | 707.23 | 598.17 ± 96.69 |
| IL-10, pg/mL | 33.06 | 76.7 | 74.31 | 61.36 ± 24.50 | 122.16 | 187.96 | 148.84 | 152.99 ± 33.09 |
